# UV irradiation to mouse skin decreases hippocampal neurogenesis and synaptic protein expression via HPA axis activation

**DOI:** 10.1038/s41598-017-15773-z

**Published:** 2017-11-14

**Authors:** Mira Han, Jae-Jun Ban, Jung-Soo Bae, Chang-Yup Shin, Dong Hun Lee, Jin Ho Chung

**Affiliations:** 1 Department of Biomedical Sciences, Seoul National University Graduate School, Seoul, South Korea; 2 Department of Dermatology, Seoul National University College of Medicine, Seoul, South Korea; 3 Institute of Human-Environment Interface Biology, Medical Research Center, Seoul National University, Seoul, South Korea; 4 Institute on Aging, Seoul National University, Seoul, 03080 Republic of Korea

## Abstract

The skin senses external environment, including ultraviolet light (UV). Hippocampus is a brain region that is responsible for memory and emotion. However, changes in hippocampus by UV irradiation to the skin have not been studied. In this study, after 2 weeks of UV irradiation to the mouse skin, we examined molecular changes related to cognitive functions in the hippocampus and activation of the hypothalamic-pituitary-adrenal (HPA) axis. UV exposure to the skin decreased doublecortin-positive immature neurons and synaptic proteins, including N-methyl-D-aspartate receptor 2 A and postsynaptic density protein-95, in the hippocampus. Moreover, we observed that UV irradiation to the skin down-regulated brain-derived neurotrophic factor expression and ERK signaling in the hippocampus, which are known to modulate neurogenesis and synaptic plasticity. The cutaneous and central HPA axes were activated by UV, which resulted in significant increases in serum levels of corticosterone. Subsequently, UV irradiation to the skin activated the glucocorticoid-signaling pathway in the hippocampal dentate gyrus. Interestingly, after 6 weeks of UV irradiation, mice showed depression-like behavior in the tail suspension test. Taken together, our data suggest that repeated UV exposure through the skin may negatively affect hippocampal neurogenesis and synaptic plasticity along with HPA axis activation.

## Introduction

Ultraviolet (UV) irradiation is essential for the synthesis of vitamin D^1^. However, excessive UV irradiation causes skin aging^2^ and skin cancer^3,4^. Depending on their wavelength, UV rays are classified as UVA (320–400 nm), UVB (280–320 nm), and UVC (100–280 nm). UVC is absorbed by the ozone layer, and therefore, does not reach the earth’s surface^5,6^. UVB is mostly absorbed by the epidermis and UVA penetrates more deeply into the dermis^7^.

The skin is the largest organ of the body and a front-line homeostatic barrier to the external environment^8,9^. External environment are sensed by the skin and transmitted to the brain^10^, and changes of the skin status can remotely modulate homeostatic brain function via systemic cytokines^11,12^. Conversely, skin condition can be changed due to neuropsychological comorbidity^13^. This skin-brain bidirectional communication regulates local and systemic homeostasis via the cutaneous neuroendocrine system, containing serotoninergic and melatoninergic systems and the hypothalamic-pituitary-adrenal (HPA) axis^9^.

Stressful stimuli trigger the activation of the HPA axis via the secretion of corticotropin-releasing hormone (CRH) in the hypothalamic paraventricular nucleus. This in turn stimulates the anterior pituitary to release adrenocorticotropic hormone (ACTH). Circulating ACTH binds to melanocortin 2 receptor (MC2R). This leads to the initiation of glucocorticoid (GC) synthesis in the adrenal cortex^14^. A fully functional HPA axis homolog called the cutaneous HPA axis exists in the skin. Various skin cells, including keratinocytes, melanocytes and outroot sheet cells, express HPA axis elements such as CRH, ACTH, GCs, and their receptors^15^. GCs, which are steroid hormones and the final products of the HPA axis, are tightly regulated by negative feedback^16^. GCs circulate in the body and bind to glucocorticoid receptors (GRs), thereby exerting various physiological actions, such as anti-inflammatory, anti-proliferative, and vasoconstrictive effects^17^. GRs are abundantly expressed in specific brain regions, including the hippocampus, prefrontal cortex, and amygdala in humans and rodents, and are regulated by GCs^18^.

The hippocampus plays an important role in emotion response and memory consolidation. Hippocampal neurogenesis and synaptic plasticity are closely related to these hippocampal functions^19^. In adult mammals, two specific brain regions show neurogenesis throughout life: the subventricular zone of the lateral ventricles and the subgranular zone (SGZ) of the dentate gyrus (DG) in the hippocampus^20^. Newly generated hippocampal neurons from neural progenitor cells (NPCs) in the SGZ can integrate into preexisting neural circuits. The proliferation, survival, and differentiation of NPCs are regulated via various neurotrophic factors and growth factors^21^. For example, exercise increases neurogenesis and neural plasticity^22^ via increased brain-derived neurotrophic factor (BDNF)^23^, insulin-like growth factor-1 (IGF-1)^24^, and vascular endothelial growth factor^25^, while stress and aging decrease neurogenesis and neurotrophic factors^26,27^.

The *N*-methyl-d-aspartate receptor (NMDAR) and α-amino-3-hydroxy-5-methyl-4-isoxazolepropionic acid receptor (AMPAR) are ion channels that are essential for synaptogenesis, experience-dependent synaptic remodeling, and synaptic efficacy^28^. Cognitive deficits occur when the hippocampal NMDARs or AMPARs are impaired, indicating that NMDAR- and AMPAR-dependent synaptic plasticity is essential for normal learning and memory^29,30^. Moreover, two synapse marker proteins, post-synaptic density protein 95 (PSD-95) and synaptophysin (SYP), are involved in synaptic signal transmission. PSD-95 plays an important role in synaptic maturation and synaptic plasticity^31^. SYP, an integral membrane protein of synaptic vesicles, reflects changes in synapse vesicles, and SYP expression is reduced by synaptic dysfunction^32^.

In stress conditions, the hippocampus undergoes various changes, including morphological changes, decreased adult neurogenesis, and modification in synaptic plasticity via increased GCs^33^. Recent studies have shown that UV irradiation is an external stressor that leads to increases in the blood levels of GCs in rodents^34^. However, it is still unknown whether UV irradiation to the skin increases GC levels and has a negative effect on the hippocampus. Therefore, we studied the adverse effects of UV irradiation of mouse skin on adult hippocampal neurogenesis and synaptic protein expression, which may play important roles in emotion-related behaviors.

## Results

### UV irradiation to mouse skin decreased adult hippocampal neurogenesis

**Figure 1:**
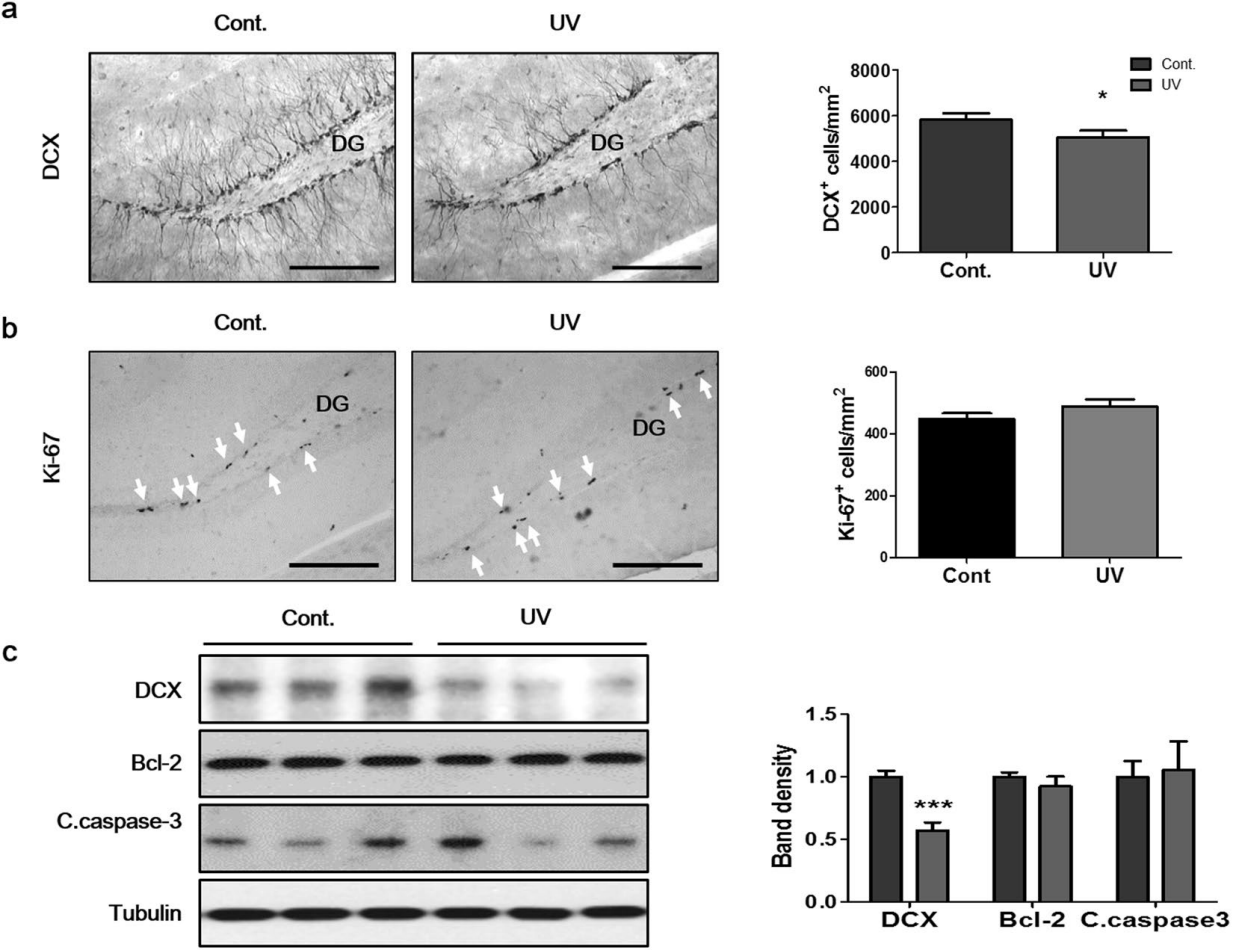
UV irradiation of mouse skin decreased adult hippocampal neurogenesis. Representative immunohistochemical images of (**a**) DCX-positive and (**b**) Ki-67-positive cells in the DG. DCX- and Ki-67-positive cells were counted in 6 hippocampal sections from each mouse and in 8 mice from each group, and mean values were calculated. (**c**) The protein expression levels of DCX, Bcl-2, and cleaved caspase-3 were assessed in hippocampal lysates using Western blot. Relative band density was analyzed using Image J software. Tubulin was used as the endogenous control. The blots are cropped raw images are provided in Supplementary Figure S2. Scale bars: 100 µm. Graphs show means ± SEM (n = 8 mice/group). *P < 0.05 and ***P < 0.001 indicate significant differences when compared to the control group. Bcl-2, B-cell lymphoma 2; C.caspase-3, cleaved caspase-3; DCX, doublecortin; DG, dentate gyrus.

To examine the effects of UV irradiation of mouse skin on hippocampal neurogenesis and neuronal survival, biomarkers for neurogenesis, proliferation, and apoptosis were analyzed after repeated UV irradiation. Immunohistochemistry revealed that 2 weeks of UV irradiation to the skin decreased the number of doublecortin (DCX)-positive immature neurons in the hippocampal DG when compared to that in the sham-irradiated group (83.6 ± 8.2% of control, p = 0.0114, Fig. 1a). However, the number of neurons positive for Ki-67, which is a proliferation marker, was not affected (Fig. 1b). Western blot results also confirmed that UV irradiation to the skin significantly decreased DCX expression in hippocampal lysates (57.2 ± 18.0% of control, p = 0.0003, Fig. 1c). The expression levels of the apoptosis-related protein Bcl-2 and cleaved caspase-3 in hippocampal lysates from UV-irradiated mice were the same as those in the control group. These results show that repeated UV exposure of the skin can lead to decreased hippocampal neurogenesis.

### UV irradiation to mouse skin reduced the levels of the synaptic proteins NMDAR2A and PSD-95 in the hippocampus

**Figure 2:**
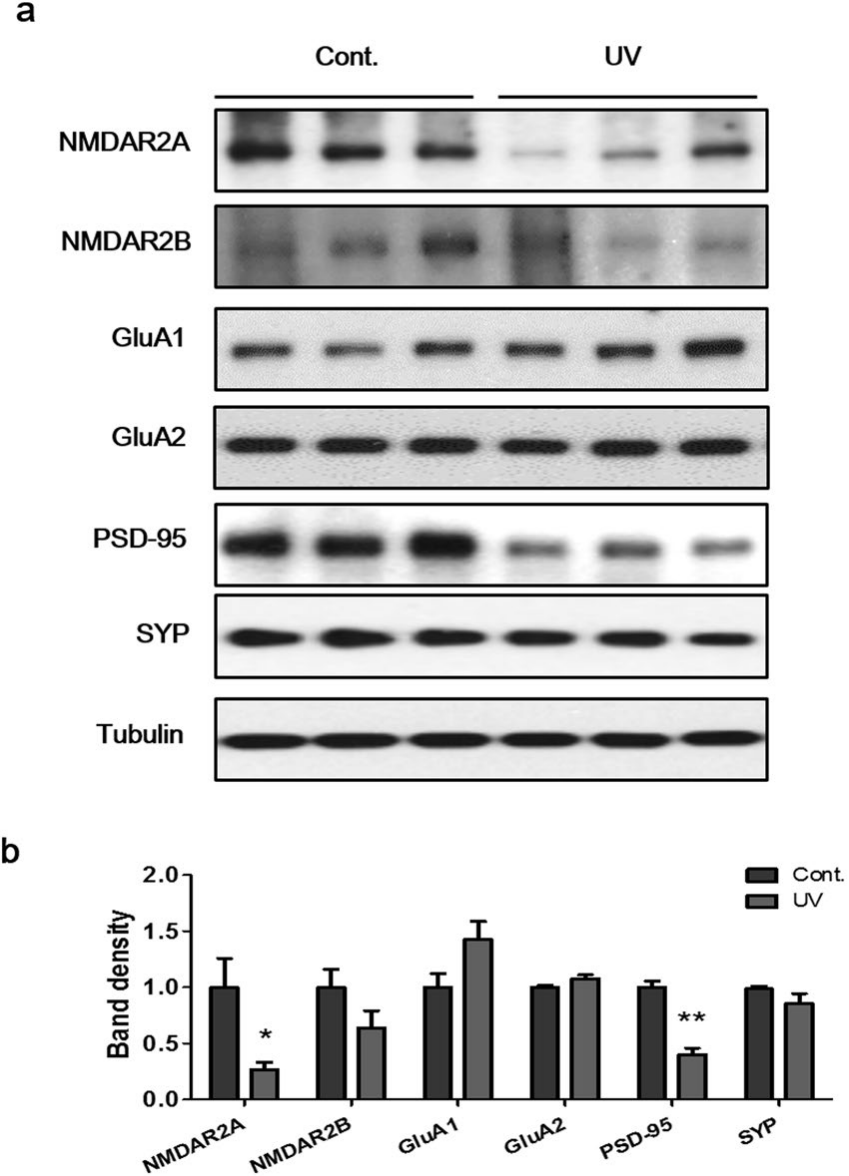
The expression levels of synaptic proteins NMDAR2A and PSD-95 were reduced by UV irradiation. (**a**) Representative western blot analysis results, and (**b**) density graphs of NMDAR2A, NMDAR2B, GluA1, GluA2, PSD-95, and SYP in the DG. Relative band density was analyzed using Image J software and normalized by tubulin. The blots are cropped raw images are provided in Supplementary Figure S3. Graphs show means ± SEM (n = 8 mice/group). *P < 0.05 and ***P < 0.001 indicate significant differences when compared to the control group. GluA1, glutamate receptor 1; GluA2, glutamate receptor 2, NMDAR2A, N-methyl-D-aspartate receptor 2 A; NMDAR2B, N-methyl-D-aspartate receptor 2B; PSD-95, post-synaptic density protein 95; SYP, synaptophysin.

To investigate the effects of UV irradiation on hippocampal synaptic plasticity, we examined the levels of synaptic proteins, including NMDAR subunits (NMDAR2A and NMDAR2B), AMPAR subunits (GluA1 and GluA2), PSD-95, and SYP in the hippocampus using Western blot (Fig. 2a). Interestingly, 2 weeks of UV irradiation to the skin decreased the hippocampal levels of NMDAR2A and PSD-95 (26.7 ± 18.6% and 40.2 ± 10.1% of control, p = 0.0148 and p = 0.0017, respectively; Fig. 2b). In contrast, the levels of other synaptic proteins did not differ from those of the sham-irradiated group. Our results indicate that UV irradiation to the skin decreases the expression of the synaptic proteins such as NMDAR2A and the complex protein PSD-95.

### UV irradiation to mouse skin suppressed the hippocampal expression of BDNF and phosphorylated ERK

**Figure 3:**
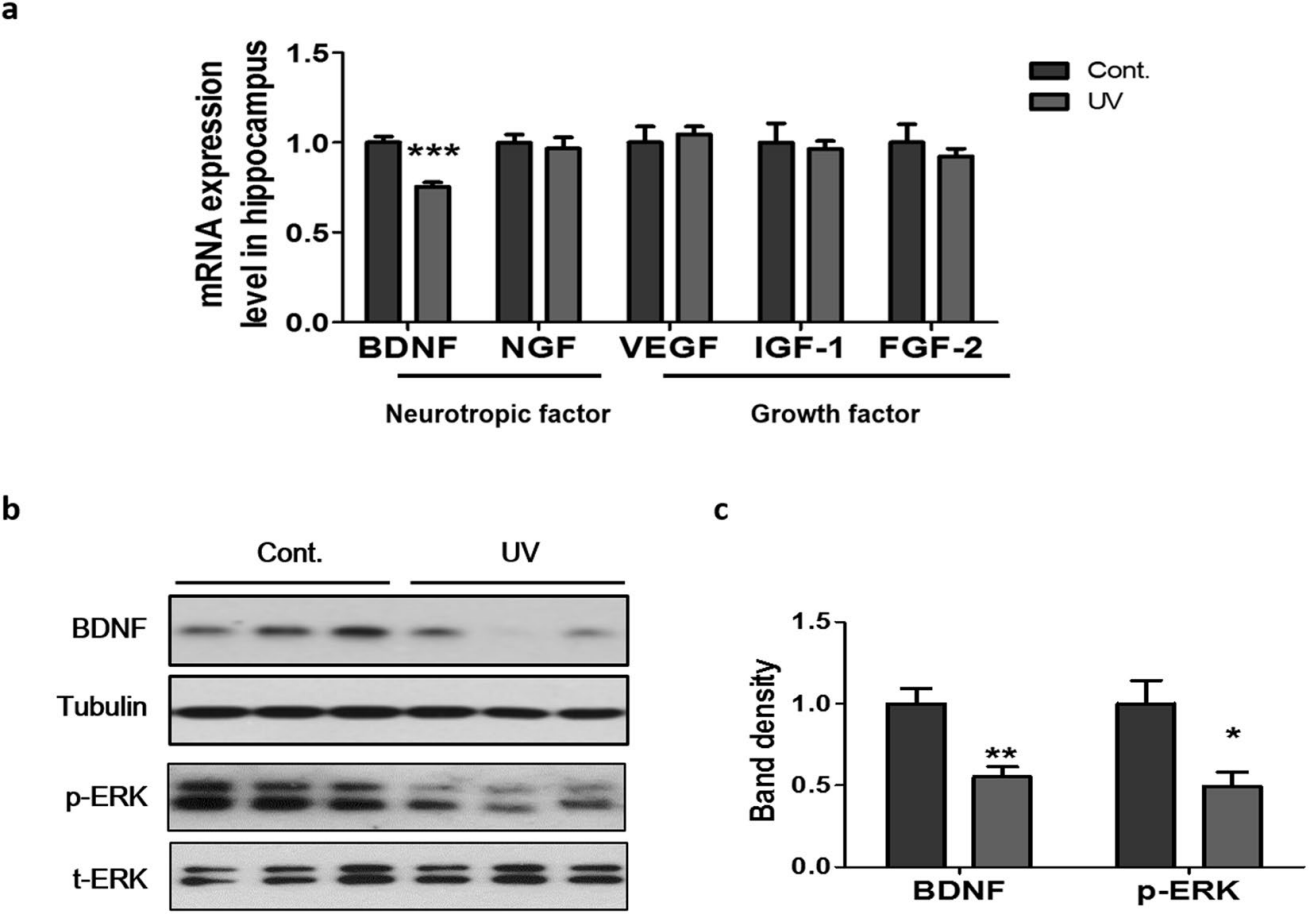
UV irradiation suppressed the expression of BDNF and phosphorylated ERK in the hippocampus. (**a**) Neurotropic factors (BDNF, NGF) and growth factors (VEGF, IGF-1, and FGF-2) mRNA expression levels in the hippocampus were analyzed using RT-qPCR. GAPDH was used to normalize the mRNA expression levels. Representative Western blot images and density graphs of (**b**) BDNF, p-ERK, and t-ERK in the hippocampus. (**c**) Relative band density was analyzed using image J software. Tubulin and t-ERK were used as endogenous controls, respectively. The blots are cropped raw images are provided in Supplementary Figure S4. Graphs show means ± SEM (n = 8mice/group). *P < 0.05, **P < 0.01, and ***P < 0.001 indicate significant differences when compared to control group. BDNF, brain-derived neurotrophic factor; FGF-2, fibroblast growth factor 2; IGF-1, insulin-like growth factor 1; NGF, nerve growth factor; p-ERK, phosphorylated extracellular signal-related kinase; t-ERK, total extracellular signal-related kinase VEGF, vascular endothelial growth factor.

To further understand how UV irradiation to the skin modulates neurogenesis and synaptic proteins in the hippocampus, we investigated expression changes in neurotropic factors such as BDNF and NGF, and growth factors such as VEGF, IGF-1, and FGF-2 in the hippocampus using RT-qPCR. Interestingly, BDNF mRNA levels were significantly down-regulated (75.4 ± 7.0% of control, p = 0.0003, Fig. 3a), while the mRNA levels of other genes were unchanged. BDNF plays critical roles in the enhancement of neurogenesis, neuroprotection, and synaptic plasticity^35^. Using Western blot analysis, we observed that skin UV exposure significantly decreased BDNF protein expression in the hippocampus (55.1 ± 18.2% of control, p = 0.0014, Fig. 3b,c). The ERK signaling pathway is activated when BDNF binds to tropomyosin-related kinase B, which is known as a BDNF receptor, and plays an important role in neuronal progenitor maturation and synaptic development^36^. Consistently, we also found that the levels of phosphorylated ERK were significantly down-regulated in the hippocampus following UV irradiation of the skin (49.5 ± 22.7% of control, p = 0.0140, Fig. 3b,c). Our results suggest that exposure of skin to UV radiation leads to down-regulation of BDNF expression and suppression of ERK activity in the hippocampus, which may result in decreased neurogenesis and synaptic protein expression in the hippocampus.

### UV irradiation to mouse skin activated the central and cutaneous HPA axes

**Figure 4:**
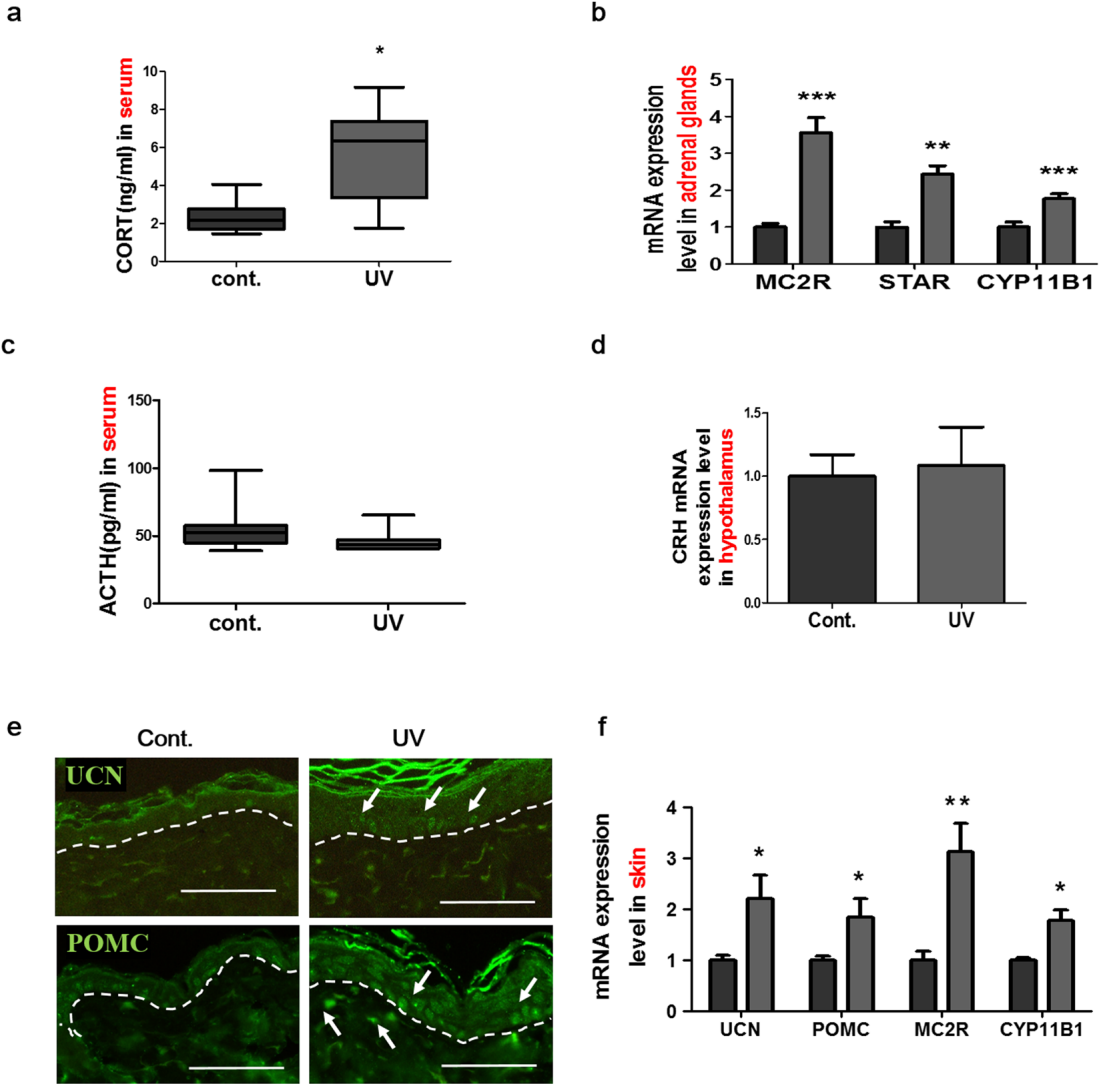
UV irradiation stimulated both central and cutaneous HPA axes. Serum levels of (**a**) CORT and (**c**) ACTH were measured using ELISA assay. (**b**) MC2R, STAR, and CYP11B1 mRNA levels in the adrenal glands, and (**d**) CRH mRNA expression levels in the hypothalamus were measured using RT-qPCR. (**e**) UCN and POMC expression levels in mouse skin with or without ultraviolet radiation exposure were examined using immunohistochemistry. Scale bars: 50 µm under 400x magnification. (**f**) The mRNA expression levels of UCN, POMC, MC2R, and CYP11B1 in the skin were analyzed using RT-qPCR. All relative mRNA levels were normalized to those of the endogenous control GAPDH. Graphs show means ± SEM (n = 8mice/group). *P < 0.05, **P < 0.01, and ***P < 0.001 indicate significant differences between groups. ACTH, adrenocorticotropic hormone; CORT, corticosterone; CRH, corticotropin-releasing hormone; CYP11B1, cytochrome P450 family 11 subfamily B member 1; MC2R, melanocortin 2 receptor; POMC, pro-opiomelanocortin; STAR, steroidogenic acute regulatory protein; UCN, urocortin.

Stress-induced GCs are the most potent neurogenesis suppressors^37^. Moreover, reduced BDNF levels have been reported in chronic corticosterone (CORT) treated rats^38^. Therefore, we investigated whether UV radiation for 2 weeks could induce HPA axis activation and whether the subsequent increase in CORT levels played a role in the UV irradiation-induced suppression of hippocampal neurogenesis and synaptic protein expression. First, we analyzed the serum levels of CORT using ELISA. CORT concentrations were markedly elevated 12 h after the last UV exposure in the sera of mice irradiated for 2 weeks (248.8 ± 106.9% of control, p = 0.0148, Fig. 4a). Next, to examine central HPA axis activation, we analyzed steroidogenesis in the adrenal glands. When ACTH binds to MC2R, adrenocortical steroidogenesis is triggered and cholesterol is transported into the mitochondria by steroidogenic acute regulatory protein (STAR). In addition, CORT levels are regulated by cytochrome P450 family 11 subfamily b member 1 (CYP11B1), which is involved in the conversion of 11- deoxycorticosterone to CORT in the adrenal cortex^39^. Interestingly, the mRNA levels of MC2R, STAR, and CYP11B1 were significantly increased (355.8 ± 114.5%, 234.0 ± 64.1%, and 177.0 ± 37.1% of control, p = 0.002, p = 0.0002, p = 0.007, respectively; Fig. 4b) in the adrenal glands of UV-irradiated mice. However, the serum level of ACTH (Fig. 4c) and CRH mRNA level in the hypothalamus (Fig. 4d) were unchanged in the UV-irradiated mice. Since the HPA axis is tightly regulated by a negative feedback mechanism^40^, the levels of ACTH and CRH in the UV-irradiated group may be due to negative feedback regulation.

The cutaneous HPA axis can contribute to an increase in the level of circulating CORT following acute UV irradiation^34^. To examine whether the cutaneous HPA axis was activated in our experimental setting, we performed immunohistochemical staining of UV-irradiated mouse skin to evaluate the expression of urocortin (UCN), which is a member of the CRH family, and that of pro-opiomelanocortin (POMC). In UV-irradiated mice, UCN expression was increased in the epidermis, and POMC expression was enhanced in both the epidermis and the dermis in UV-irradiated mice (Fig. 4e). Consistently, mRNA levels of UCN, POMC, MC2R, and CYP11B1 were up-regulated (221.2 ± 121.7%, 184.6 ± 97.9%, 313.0 ± 147.6%, and 178.5 ± 53.1% of control, p = 0.0175, p = 0.0262, p = 0.0041, and p = 0.0111, respectively; Fig. 4f). Our results indicate that repeated UV irradiation to the skin increases the levels of circulating CORT via the activation of the central and cutaneous HPA axes.

### UV irradiation of mouse skin induced glucocorticoid receptor activation in the hippocampus

**Figure 5:**
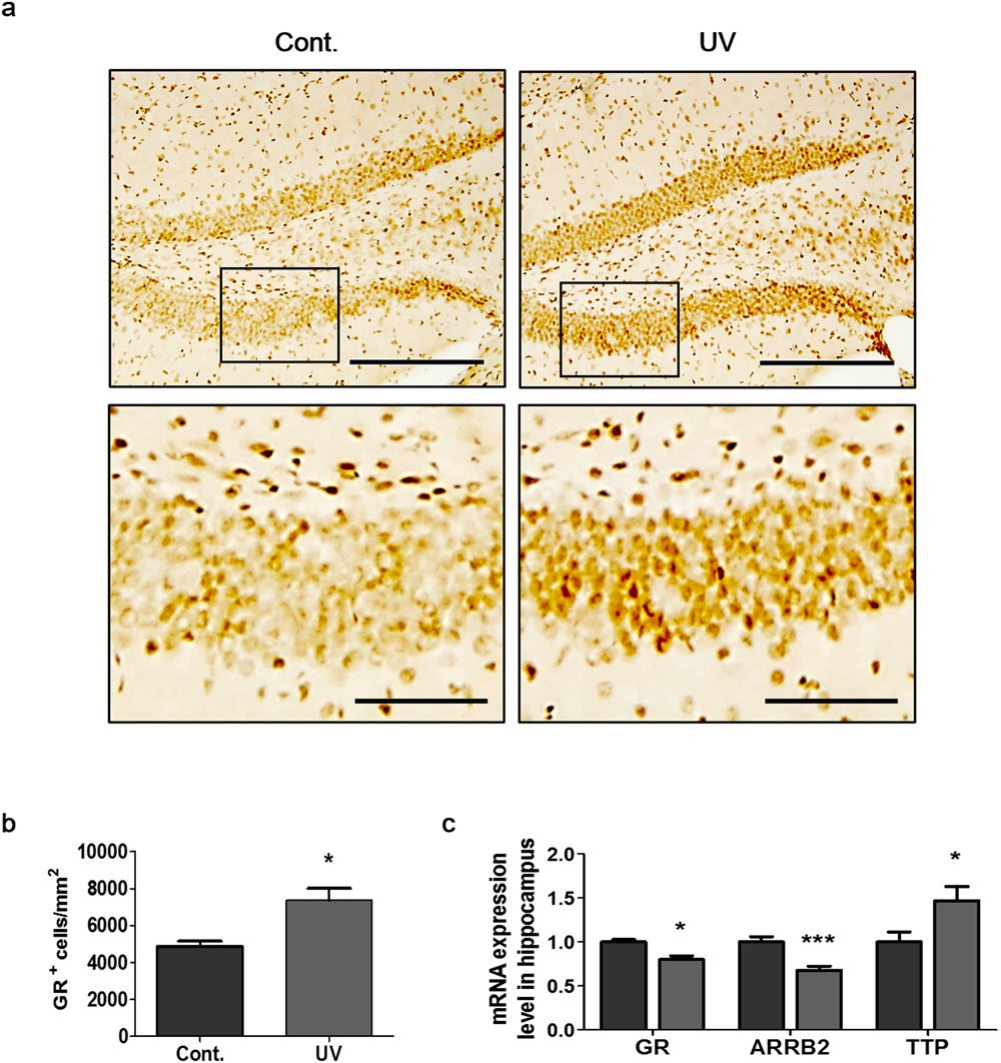
UV irradiation of mouse skin induced nuclear translocation of GR and modulated the expression levels of its target genes. (**a**) Representative immunohistochemical images of GR-positive cells under 200x (upper panel) and high magnification (lower panel) of insert area in the hippocampus. Scale bars: 100 µm and 50 µm, respectively. The graphs show (**b**) GR-positive cell numbers counted in 8 brain sections from each mouse. (**c**) Relative GR, ARRB2, and TTP mRNA expression levels in the hippocampus were analyzed using RT-qPCR and normalized to GAPDH expression levels. Graphs show means ± SEM (n = 8mice/group). *P < 0.05 and ***P < 0.001 indicate significant differences between groups. ARRB2, arrestin beta 2; GR, glucocorticoid receptor; TTP, tristetraproline.

To confirm whether the CORT produced following UV irradiation binds to GRs in the hippocampus, we quantified GR staining in the DG (Fig. 5a). GR is translocated to the nucleus when it is bound by GCs, and acts as a transcription factor^41^. Our results showed that the numbers of cells with GR-positive nuclei in the DG were markedly increased in the UV-irradiated group (149.7 ± 42.6% of control, p = 0.0411, Fig. 5b). Expression of both GR and arrestin beta 2 (ARRB2) is down-regulated following GR activation^42^, while tristetraprolin (TTP) expression is up-regulated^43^. We thus analyzed the expression of GR target genes in the hippocampus. Our results revealed that exposure of the skin to UV radiation significantly reduced GR and ARRB2 mRNA levels and increased TTP mRNA levels in the hippocampus (79.9 ± 12.5%, 67.8 ± 12.5%, and 146.8 ± 45.4% of control, p = 0.0030, p = 0.0006, and p = 0.0370, respectively; Fig. 5c). Our findings suggest that UV irradiation of the skin increases circulating CORT, which may then bind to hippocampal GRs and lead to decreases in hippocampal neurogenesis and synaptic proteins expression.

### UV irradiation of mouse skin caused depression-like behavior

**Figure 6:**
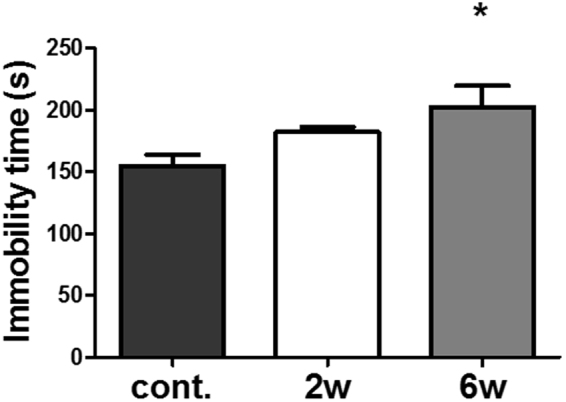
UV irradiation of mouse skin results in depression-like behavior. To assess a depression-like phenotype (immobility) in UV-irradiated mice, we performed the tail suspension test. UV light was applied to the dorsal skin under anesthesia 3 days a week for 2 or 6 weeks, and the immobility time was measured during the 6 min test session and compared with that of sham-irradiated mice. Graphs show means ± SEM (n = 8 mice/group). *P < 0.05, indicate significant differences compared with control group values. Cont., Sham-UV irradiated group; 2w, 2 weeks UV-irradiated group; 6w, 6 weeks UV-irradiated group.

Deficits in adult hippocampal neurogenesis and synaptic plasticity in rodents affect the regulation of mood and spatial memory^19,44^. To investigate whether UV-irradiation to the skin leads to hippocampus-dependent behavioral changes, we performed the tail suspension test (TST). As 2 weeks of UV irradiation was found to be insufficient for producing behavioral change, a 6-week long UV irradiation period was used. A significantly increased immobility time was observed in mice chronically exposed to UV radiation for 6 weeks (129.8 ± 24.4% of control, p = 0.0099, Fig. 6), indicating that chronic UV-irradiation may cause depression-like behavior. Interestingly, only the number of DCX-positive cells was reduced in the 2-week group, while the number of both DCX- and Ki-67-positive cells were decreased in the 6-week group (Supplementary Figure S1). In this experimental condition, UV exposure did not affect memory, as measured using the Y-maze and novel object recognition tests (data was not shown). Thus, our results indicate that chronic excessive UV-irradiation affects hippocampal function in mice.

## Discussion

Excessive UV irradiation is considered to be the main cause of skin aging^45^ and skin cancer^4^. In addition, chronic UV irradiation results in addiction to UV light due to β-endorphin production^46^. However, there have been no reports of changes in the hippocampus following repeated exposure of the skin to UV radiation. Here, we report for the first time that repeated exposure of the skin to UV radiation leads to a stress response affecting the hippocampus.

During adult neurogenesis, DCX is expressed in the NPCs. The expression of this protein is down-regulated when the cells begin to express NeuN, which is a marker of mature neurons. Thus, DCX-positive cells are considered as developing neurons^47^. Various studies have shown that different stressors have different effects on hippocampal neurogenesis because neurogenesis is a multi-step process^48–51^. A large number of NPCs undergo programmed cell death after proliferation during a fine-tuning process^52^. Some stressors, such as predator odor, maternal deprivation, and psychosocial stress stimuli only reduce the numbers of DCX-positive cells without altering their proliferation^53–55^. We found decreased numbers of DCX-positive cells in the SGZ after 2 weeks of UV irradiation, while NPC proliferation and apoptosis were unchanged. Thus, we can deduce that UV radiation inhibits hippocampal neuronal development.

In this study, we observed decreases in synaptic proteins in the UV-irradiated mice hippocampus, such as NMDAR2A and PSD-95. Although each glutamate receptor has a different function, all mediate active signal transduction at synapses. NMDARs are closely associated with the generation of long-term potentiation, a persistent enhancement of synaptic strength by repeated high-frequency stimulation, which is involved in memory formation and synaptic plasticity^56,57^. PSD-95 is co-localized with NMDARs at excitatory synapses and modulates NMDAR channel gating and surface expression^58^. Therefore, dysfunction of NMDARs and PSD-95 is closely associated with depressive disorder^59,60^. We hypothesize that UV irradiation of the skin affects hippocampal functions by modulating the expression of synaptic protein such as NMDAR2A and PSD-95 and by inhibiting hippocampal neurogenesis.

BDNF is the most abundant neurotrophin in the brain^61^. BDNF levels are reduced in patients and in animal models of depression^62,63^, and antidepressant drugs increase BDNF levels in the brain^64,65^. Considering these important roles of BDNF in depression, we examined hippocampal BDNF levels in our model. We observed that hippocampal BDNF and phosphorylated ERK expression was significantly decreased by UV irradiation of the skin. This suggests that UV irradiation affects hippocampus-dependent functions via deficits in neurogenesis, synaptic plasticity, and BDNF expression.

Because of the skin and hippocampus are separated, we hypothesized that CORT, a hormone that negatively affects the hippocampus, acts as a signaling mediator between the skin and the hippocampus. We observed UV irradiation-induced increases in CORT levels in the systemic circulation. An elevated circulating CORT level is a common marker of stress and leads to the suppression of adult neurogenesis. Consistent with the findings in rodents, patients with Cushing’s disease, which leads to excessive secretion of cortisol, have depressive symptoms^66^ and memory deficits^67^. Our results demonstrated that the levels of both MC2R and steroidogenesis-related enzyme were up-regulated in the adrenal gland as a stress response after UV irradiation. These results indicated that the UV-induced CORT level increase is due to adrenal gland activation. We found up-regulation of molecules associated not only with the central HPA axis, but also with the cutaneous HPA axis, including UCN, POMC, MC2R, and CYP11B1. Hair follicles also synthesize HPA axis components, and this synthesis varies according to the hair cycle stage^68^. To exclude change in the cutaneous HPA axis due to variation in the hair cycle stage, we used 6– and 12–week old mice at the telogen stage^69^. Our results suggest that UV may affect the hippocampus via changes in CORT levels in the skin and adrenal glands.

Abundant evidence indicates that GRs activated by the binding of GCs adversely affect hippocampal neurogenesis, synaptic plasticity, and memory function^70–72^. The direct mechanism of action of GRs involves their dimerization and translocation into the nucleus, where they bind to specific DNA responsive elements, which in turn activate gene expression in a process called transactivation^73^. Our immunohistochemical staining results confirmed that the number of cells with GR-positive nuclei was higher in the UV-irradiated group than in the non-irradiated group, and that the levels of GR target genes, such as GR, ARRB2, and TTP were modulated after UV irradiation. These results indicate that UV irradiation of the skin led to a stress response, which in turn activated GR dependent signaling pathways in the hippocampus.

In addition to the increase in CORT in UV-irradiated mice, increased inflammatory cytokine expression may also affect brain function. Increased levels of systemic inflammatory cytokines in an animal model of psoriasis have been shown to affect brain homeostasis, leading to depressive behavior^11,12^. Further investigation is needed to understand the additional mechanisms of UV radiation effects on brain function.

The TST is an appropriate behavioral assessment tool for evaluating depression-like phenotypes^74^. LPS-injected mice show increased immobility time in the TST, which is a characteristic of depression^75^. In this study, we did not observe depression-like behavior after 2 weeks of UV irradiation, although hippocampal molecular levels such as BDNF, DCX, NMDAR2A, and PSD-95 were modulated. Therefore, we investigated whether chronic UV exposure could alter behavior. Results of TST conducted after 6 weeks of UV irradiation indicated a statistically significant increase in immobility time. In addition, hippocampal neurogenesis was more severely suppressed in mice irradiated with UV for 6 weeks than in mice irradiated for 2 weeks. These data indicate that excessive repeated UV irradiation to the skin results in hippocampal damage and depressive behavior.

Gamma rays induce cognitive deficit by directly damaging NPCs^76^. Ablation of neurogenesis has been also demonstrated in mice irradiated with X-rays^77,78^. These two rays have strong penetration ability and damage cells directly. Unlike these ionizing radiations with high penetration power, most UVB rays are absorbed in the epidermis, and UVA rays are absorbed in the dermis. In this study, we demonstrated that the inhibitory effects of UV radiation on hippocampal neurogenesis and synaptic plasticity are due to indirect effects of UV radiation through activation of the HPA axis.

In conclusion, we report for the first time that chronic UV irradiation of the skin can decrease hippocampal neurogenesis and synaptic signaling, leading to mood changes. We propose that these changes are caused by UV-induced activation of central and cutaneous HPA axis.

## Methods

### Animals and UV irradiation

All experimental protocols were approved by the Institutional Animal Care and Use Committee (Case Number: 16–0072-S1A0) of the Biomedical Research Institute at Seoul National University Hospital and were performed in accordance with relevant guidelines and regulations. Female C57BL/6 mice were purchased from Orient Experimental Animal Breeding Center (Seoul, Korea) at 5 weeks of age. All animals were housed, 4 per cage, under standard controlled room conditions with food and water available *ad libitum*. The skin on the backs of the mice was shaved, 2 days prior to UV irradiation using electric clippers, under anesthesia (4% isoflurane). Irradiation was performed using TL20W/12RS UV lamps (Philips, Eindhoven, The Netherlands) with an emission spectrum between 275 and 320 nm. UVC (<290 nm) wavelengths was blocked using Kodacel filter (TA401/407; Kodak, Rochester, NY), which was placed 2 cm in front of UV lamp. UV intensity was measured using a UV meter (model 585100, Waldmann, Villingen-Schwenningen, Germany). In the 2 week irradiation treatment protocol, 200 mJ/cm^2^ of UV light was applied to the dorsal skin under anesthesia 3 days per week (Monday, Wednesday, and Friday). Chronic excessive UV irradiation (6 week-irradiation treatment) was conducted with 200 mJ/cm^2^ for the first 2 weeks, 300 mJ/cm^2^ for the next 2 weeks, and 400 mJ/cm^2^ for the last 2 weeks.

### Immunohistochemistry

Immediately after anesthesia, the dorsal skin^79^ and brain^80^ were collected and fixed using 4% formalin for histological studies, as previously reported.

### Brain

The brain was removed 12 h after the last UV irradiation, and the left hemisphere was placed in 4% paraformaldehyde overnight, then transferred to a 30% sucrose solution, and kept at 4 °C until the brain tissue was submerged. The brain was embedded in Tissue-Tek OCT compound (Sakura Finetech, Tokyo, Japan) and stored at −80 °C. The brain tissue was sectioned coronally at 40 μm thickness with a freezing microtome; every fifth section was collected and placed in a separate well of a 24-well plate. The brain slice containing the hippocampus was mounted onto silanized slides (Dako, Carpinteria, CA, USA) and dried for 1 h. For immunostaining, the brain slides were rinsed with PBS twice and steamed for antigen retrieval. The sections were washed with PBS and blocked with UltraVision Protein Block (Thermo Scientific, Fremont, USA) for 10 min at room temperature. The slices were incubated overnight at 4 °C with primary antibodies in a diluent buffer containing 1% bovine serum albumin (Sigma-Aldrich, MO, USA) and 1% Triton X-100 in 0.1 M phosphate buffer. After subsequent washing in PBS, the sections were incubated for 1 h at 4 °C with secondary antibody, then incubated with a Vector ABC kit (Vector Laboratories Ltd., CA, USA) and the reaction was visualized with 3,30-diaminobenzidine (DAB; Vector Laboratories Ltd.). The images were acquired with a Leica DM5500B microscope (Leica Microsystems, Wetzlar, Germany). For quantification of the total number of cells in the granular and subgranular zones of the dentate gyrus, the sections were coded, and the counting was performed by an examiner blinded to group allocations.

### Skin

The dorsal skin specimens were fixed in 4% paraformaldehyde, embedded in paraffin, and cut into 4 μm thick sections. The sections were stained as described above. For immunofluorescence staining, fluorescence conjugated antibodies were incubated for 1 h at room temperature and the cell nuclei was counterstained with 4′, 6-diamidino-2-phenylindole (DAPI). The images were acquired with a Leica DM5500B microscope (Leica Microsystems).

### Immunoblotting analysis

Immunoblotting was performed as previously described^80^. Briefly, proteins in hippocampal lysates were separated using SDS-PAGE and transferred to membranes. The membranes were blocked and incubated with the appropriate primary antibodies (Supplementary Table S1). After incubation with secondary antibody, the immune complexes were visualized using an ECL detection system (GE Healthcare, Buckinghamshire, UK).

### ELISA

The mice were sacrificed and blood was immediately collected in serum separator tubes (BD Microtainer Tubes, Franklin Lakes, NJ). Serum was obtained by centrifugation at 3,500 g for 20 min at 4 °C. The serum was snap-frozen in liquid nitrogen and then stored at −70 °C until the time of the measurements. The serum was thawed and diluted 1:100 to measure CORT and 1:15 to measure ACTH. We performed the assay according to the manufacturers’ protocols using commercially available kits (Supplementary Table S2).

### RNA extraction and RT-qPCR

Total RNA was extracted from the dorsal skin, adrenal glands, hippocampus, and hypothalamus using RNAiso Plus (Takara Bio Inc., Shiga, Japan). Total RNA (2 μg) was reverse transcribed to cDNA and RT-qPCR was performed using the appropriate primers (Supplementary Table S3) as described previously^80^.

### Tail-suspension test (TST)

Mice were individually suspended by the tail, using adhesive tape, at least 20 cm above the surface. At each test session, movements of the mice were recorded for subsequent analysis. Immobility during the entire 6 min test session was measured as previously validated^81^. Immobility time (depression-like behavior) was defined as the time when the animal stopped struggling and was completely motionless. Data analysis was performed by two trained researchers blinded to group allocations.

### Statistical analysis

Statistical analyses were performed using SPSS 22.0 software (IBM, Armonk, NY). Differences among groups were analyzed using Mann-Whitney tests. P-values < 0.05 were considered statistically significant.

## Electronic supplementary material

Supplementary material and figures
