# Supplementary material for: UV irradiation to mouse skin decreases hippocampal neurogenesis and synaptic protein expression via HPA axis activation
Source: Sci Rep. 2017 Nov 14;7:15574. doi: 10.1038/s41598-017-15773-z (PMC5686175; doi:10.1038/s41598-017-15773-z)
Supplement: Supplementary file 1 — Supplementary material and figures [file 41598_2017_15773_MOESM1_ESM.doc]

**UV irradiation to mouse skin decreases hippocampal neurogenesis and synaptic proteins expression via HPA axis activation**

**Mira Han^1,2,3,4+^, Jae-Jun Ban^2,3,4+^, Jung-Soo Bae^1,2,3,4+^, Chang-Yup Shin^2,3^, Dong Hun Lee^2,3,4^, and Jin Ho Chung^1,2,3,4^***

^1^ Department of Biomedical Sciences, Seoul National University Graduate School

^2^ Department of Dermatology, Seoul National University College of Medicine

^3^ Institute of Human-Environment Interface Biology, Medical Research Center, Seoul National University

^4^Institute on Aging, Seoul National University, Seoul, 03080, Republic of Korea

* jhchung@snu.ac.kr

^+^ These authors contributed equally to this work.

**Supplementary material**

**Supplementary Figure S1.**

**
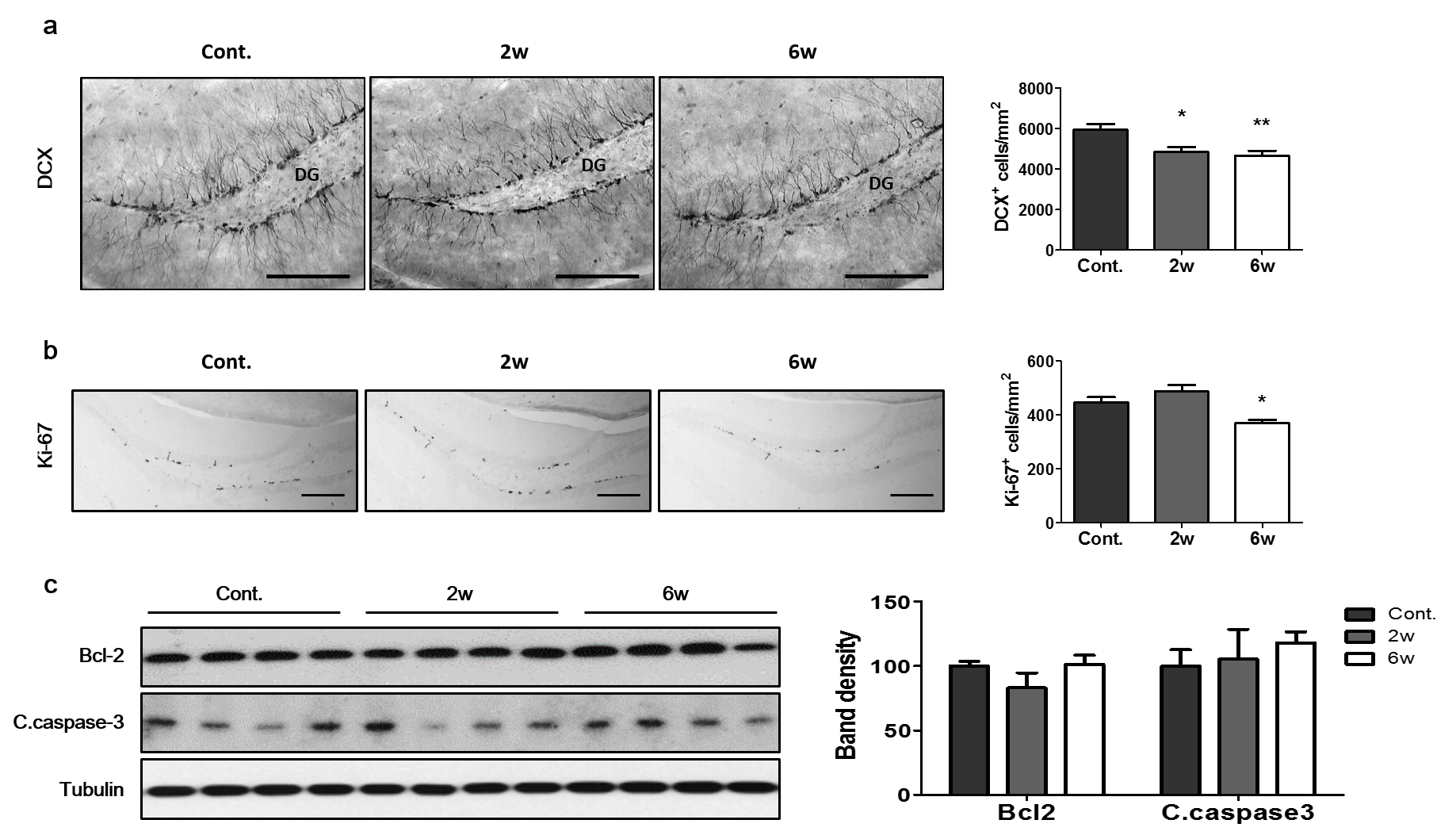
**

**Figure S1. Decreased hippocampal neurogenesis following 2 or 6 weeks of UV irradiation of the skin**

Representative images of (**a**) DCX-positive and (**b**) Ki-67-positive cells in the DG (left panel, Scale bars: 100 µm). DCX- and Ki-67-positive cells were counted in 6 hippocampal sections from each mouse and mean values were calculated. (**c**) Apoptosis related markers were assessed in hippocampal lysates using Western blot. Relative band intensities were analyzed using Image J software, and tubulin was used as the endogenous control. Graphs show means ± SEM (n = 8 mice/group). **P* < 0.05 and ****P* < 0.001 indicate significant differences when compared to the control group. Bcl-2, B-cell lymphoma 2; C.caspase-3. Cleaved caspase-3; Cont., Sham-UV irradiated group; DCX, doublecortin; DG, dentate gyrus; 2w, 2 weeks UV irradiated group; 6w, 6 weeks UV irradiated group.

**Supplementary Figure S2.**

**
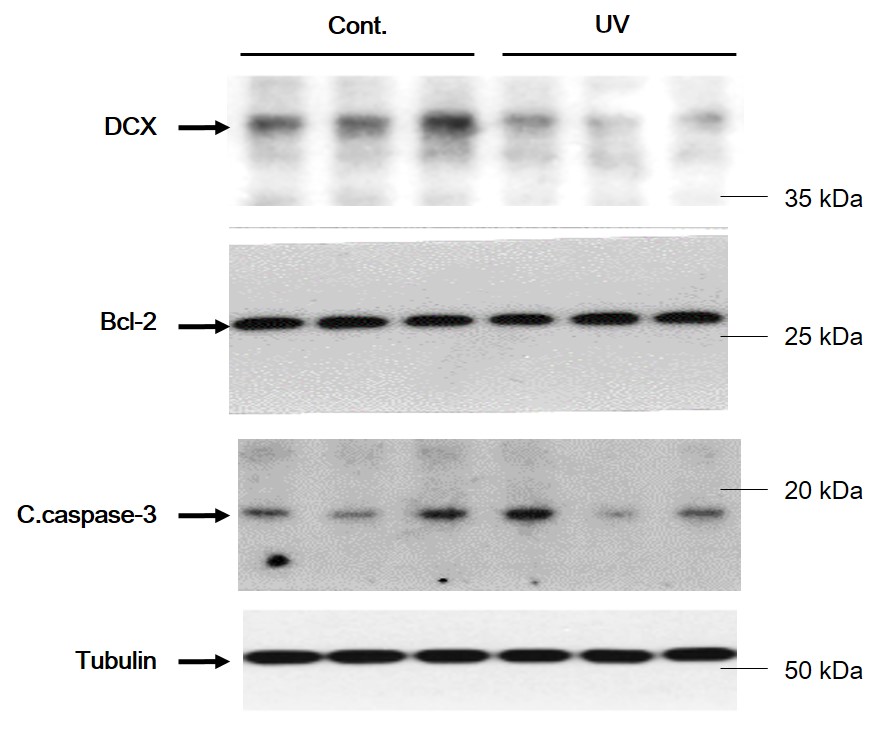
**

**Figure S2. Raw image of cropped blots in figure 1c**

**Supplementary Figure S3.**


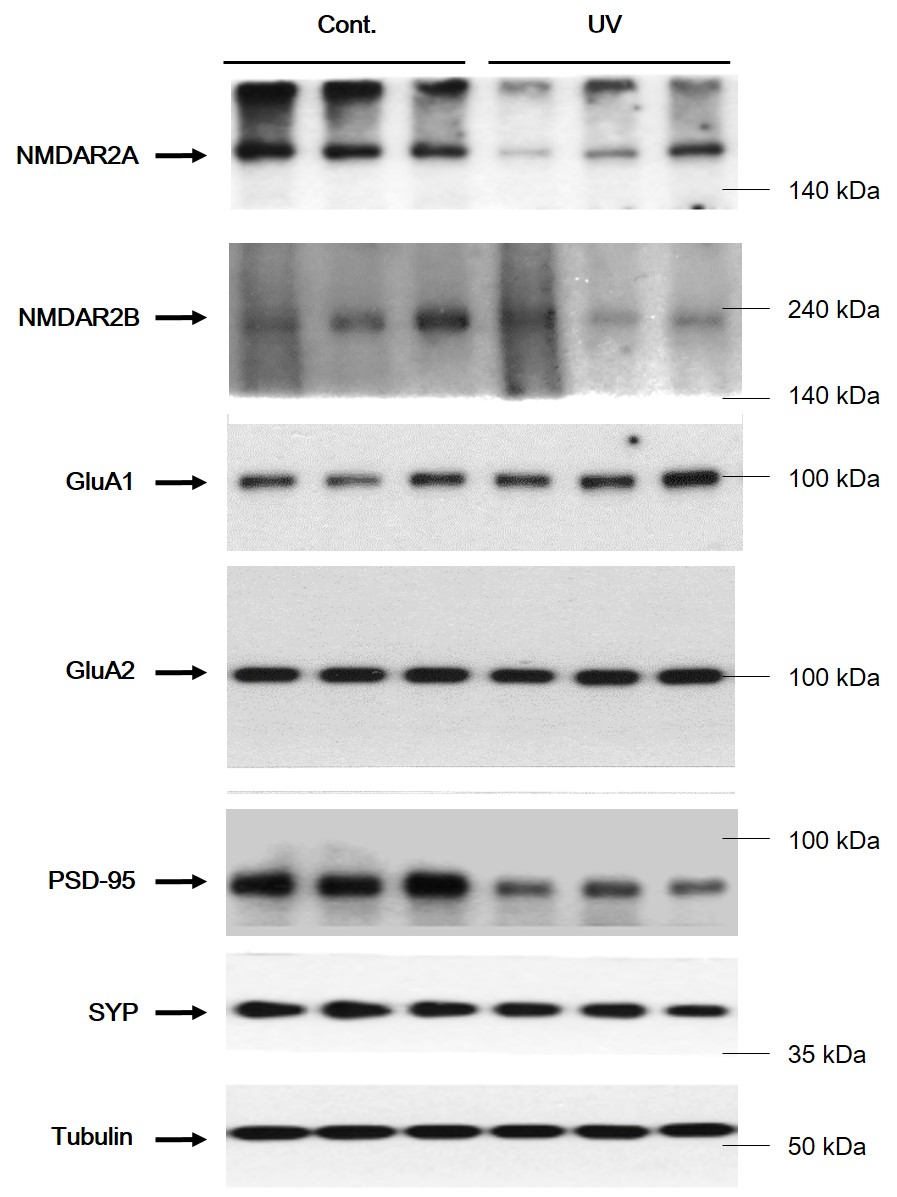


**Figure S3. Raw image of cropped blots in figure 2a**

**Supplementary Figure S4.**

**
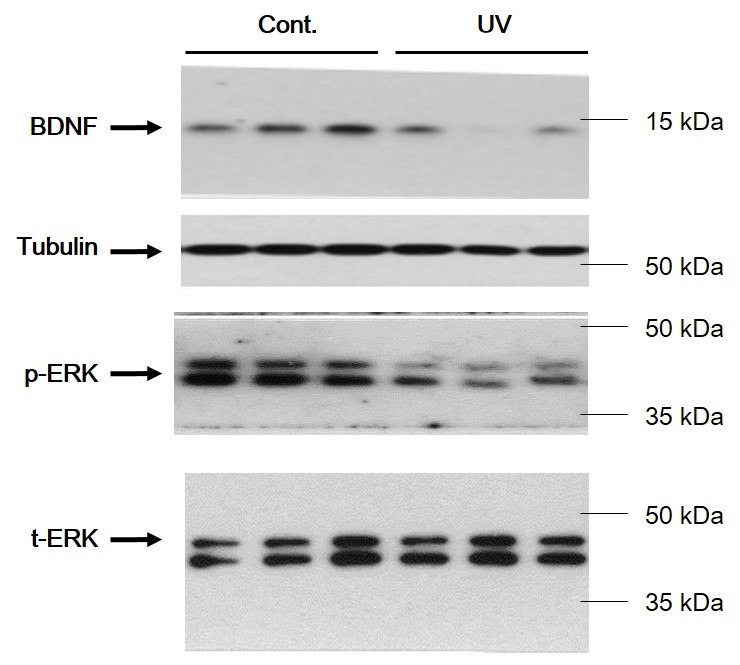
**

**Figure S4. Raw image of cropped blots in figure 3a**

Supplementary Table S1. List of primary antibodies used in this study.

| **Primary antibody** | **host** | **Cat #** | **vendor** | **Use** |
| --- | --- | --- | --- | --- |
| Doublecortin (C-18) | goat | sc-8066 | Santa Cruz Biotechnologies, Santa Cruz, CA | Immunoblotting |
| NR2B | mouse | MAB5778 | Millipore, Billerica, MA | Immunoblotting |
| GluR-1(E-6) | mouse | sc-13152 | Santa Cruz Biotechnologies, Santa Cruz, CA | Immunoblotting |
| Synaptophysin | mouse | s5768 | Sigma-Aldrich, St. Louis, MO | Immunoblotting |
| VEGF (C1) | mouse | sc-7269 | Santa Cruz Biotechnologies, Santa Cruz, CA | Immunoblotting |
| NR2A | rabbit | ab1555 | Millipore, Billerica, MA | Immunoblotting |
| GluR-2 | rabbit | AB1768-I | Millipore, Billerica, MA | Immunoblotting |
| PSD-95 | rabbit | MA1-046 | Thermo Fisher Scientific, Bremen, Germany | Immunoblotting |
| BDNF | rabbit | 3160-1 | Epitomics, Burlingame, CA | Immunoblotting |
| Bcl-2 (50E3) | rabbit | 2870 | Cell Signaling Technology, Danvers, MA | Immunoblotting |
| Cleaved Caspase-3 (Asp175) (5A1E) | rabbit | 9664 | Cell Signaling Technology, Danvers, MA | Immunoblotting |
| Phospho-Erk (Thr202/Tyr204) | rabbit | 9101 | Cell Signaling Technology, Danvers, MA | Immunoblotting |
| Erk | rabbit | 9102 | Cell Signaling Technology, Danvers, MA | Immunoblotting |
| Urocortin | goat | sc-1825 | Santa Cruz Biotechnologies, Santa Cruz, CA | Immunoblotting |
| POMC | rabbit | H-029-30 | Phenix pharmaceuticals, Burlingame, CA | Immunohistochemistry |
| Glucocorticoid Receptor (D8H2) | rabbit | 3660 | Cell Signaling Technology, Danvers, MA | Immunohistochemistry |
| **Secondary antibody** | **host** | **Cat #** | **vendor** | **Use** |
| anti-goat IgG-HRP | donkey | sc-2020 | Santa Cruz Biotechnologies, Santa Cruz, CA | Immunoblotting |
| anti-mouse IgG-HRP | goat | sc-2005 | Santa Cruz Biotechnologies, Santa Cruz, CA | Immunoblotting |
| anti-rabbit IgG-HRP | goat | sc-2004 | Santa Cruz Biotechnologies, Santa Cruz, CA | Immunoblotting |
| anti- mouse IgG Alexa 488 | goat | A11001 | Thermo Fisher Scientific, Bremen, Germany | Immunohistochemistry |
| anti-rabbit IgG Alexa 488 | goat | A11008 | Thermo Fisher Scientific, Bremen, Germany | Immunohistochemistry |

Supplementary Table S2. List of commercially available kit used in this study.

| **Target** | **Cat #** | **Vendor** |
| --- | --- | --- |
| Corticosterone ELISA kit | ADI-900-097 | Enzo Life Science, Farmingdale, NY |
| ACTH EIA kit | EK-001-21 | Phenix pharmaceuticals, Burlingame, CA |

Supplementary Table S3. Mouse primer sequences used in this study.

| **Target** | **Symbol** | **Sequence** | |
| --- | --- | --- | --- |
| Steroidogenic Acute Regulatory Protein | STAR | Forward: | TTGGGCATACTCAACAACCA |
|  |  | Reverse: | GAAACACCTTGCCCACATCT |
| Steroid 11-β-Hydroxylase | CYP11B1 | Forward: | GTATCGAGAGCTGGCAGAGG |
|  |  | Reverse: | GGGTTGATGTCGTGTCAGTG |
| Melanocortin 2 Receptor | MC2R | Forward: | TCGTGGCAGTTTTGAAAGC |
|  |  | Reverse: | TGGTGATGTAACGGTCAGCT |
| Corticotropin Releasing Hormone | CRH | Forward: | GAATTTCTTGCAGCCGGAGC |
|  |  | Reverse: | CAGCGGGACTTCTGTTGAGA |
| Urocortin | UCN | Forward: | CTG TCC ATC GAC CTC ACC TT |
|  |  | Reverse: | TCG AAT ATG ATG CGG TTC TG |
| Proopiomelanocortin | POMC | Forward: | CAT TAG GCT TGG AGC AGG TC |
|  |  | Reverse: | CTT CTC GGA GGT CAT GAA GC |
| Glucocorticoid Receptor | GR | Forward: | ACAGACTTTCGGCTTCTGGA |
|  |  | Reverse: | AAACTCCTTCTCTGTCGGGG |
| Brain Derived Neurotrophic Factor | BDNF | Forward: | TTG TTT TGT GCC GTT TAC CA |
|  |  | Reverse: | GGT AAG AGA GCC AGC CAC TG |
| Vascular Endothelial Growth Factor | VEGF | Forward: | GGACCCTGGCTTTACTGCTG |
|  |  | Reverse: | TGATTTTCTGGCTTTGTTCT |
| Glyceraldehyde-3-Phosphate Dehydrogenase | GAPDH | Forward: | AAC TTT GGC ATT GTG GAA GG |
|  |  | Reverse: | ACA CAT TGG GGG TAG GAA CA |
